# Impact of dioxins on reproductive health in female mammals

**DOI:** 10.3389/ftox.2024.1392257

**Published:** 2024-05-07

**Authors:** Nour Aldeli, Denis Murphy, Abdulsamie Hanano

**Affiliations:** ^1^ Department of Animal Biology, Faculty of Science, Al Furat University, Deir-ez-Zor, Syria; ^2^ School of Applied Sciences, University of South Wales, Cardiff, Wales, United Kingdom; ^3^ Department of Molecular Biology and Biotechnology, Atomic Energy Commission of Syria (AECS), Damascus, Syria

**Keywords:** dioxin, female reproductive system, AhR, ovary, TCDD

## Abstract

Extensive research has been conducted to investigate the toxicological impact of dioxins on mammals, revealing profound effects on the female reproductive system in both humans and animals. Dioxin exposure significantly disrupts the intricate functions of the ovary, a pivotal organ responsible for reproductive and endocrine processes. This disruption manifests as infertility, premature ovarian failure, and disturbances in sex steroid hormone levels. Comprehensive studies, encompassing accidental human exposure and experimental animal data, have raised a wealth of information with consistent yet varied conclusion influenced by experimental factors. This review begins by providing an overarching background on the ovary, emphasizing its fundamental role in reproductive health, particularly in ovarian steroidogenesis and hormone receptor regulation. Subsequently, a detailed examination of the Aryl hydrocarbon Receptor (AhR) and its role in governing ovarian function is presented. The review then outlines the sources and toxicity of dioxins, with a specific focus on AhR involvement in mediating reproductive toxicity in mammals. Within this context, the impact of dioxins, notably 2,3,7,8-tetrachlorodibenzo-p-dioxin (TCDD), on Folliculogenesis and Preimplantation embryos is discussed. Furthermore, the review delves into the disruptions of the female hormonal system caused by TCDD and their ramifications in endometriosis. Notably, variations in the effects of TCDD on the female reproductive and hormonal system are highlighted in relation to TCDD dose, animal species, and age. As a forward-looking perspective, questions arise regarding the potential involvement of molecular mechanisms beyond AhR in mediating the female reproductive toxicity of dioxins.

## 1 Introduction

The female reproductive system comprises vital structures, including the ovaries, fallopian tubes (oviducts), uterus, and vagina. This intricately organized system is hormonally regulated by the hypothalamus and pituitary. The harmonious functioning of these organs is essential for fulfilling the primary roles of the female reproductive system, which involve the production of female sex hormones, the generation of female gametes, and the transportation of these gametes to a site where they can potentially be fertilized by sperm. Following fertilization, the female reproductive system establishes an optimal environment for the development of the embryo, ultimately leading to the delivery of the fetus. Hence, the intricate functions of the female reproductive system rely on the effective operation of each individual organ (Li et al., 1995).

There is now a well-established acknowledgment that exposure to toxic chemicals can significantly impact the functioning of the female reproductive system across various levels, including the hypothalamus, pituitary gland, ovary, and reproductive tract (Crain et al., 2008).

Dysfunction in any of these organs can ultimately manifest as a disruption of ovarian function, resulting in infertility (Pocar et al., 2003). A significant number of environmental pollutants are recognized as endocrine-disrupting chemicals (EDCs) (Craig et al., 2011). As per the Environmental Protection Agency (EPA), Endocrine Disrupting Chemicals (EDCs) refer to external agents that disrupt the synthesis, secretion, transport, metabolism, binding, or elimination of naturally occurring hormones in the body, crucial for maintaining homeostasis, reproductive functions, and developmental processes (EPA, 2024) EDCs, such as bisphenol A (BPA), phthalates, polychlorinated biphenyls (PCB), dioxins, fungicides (vinclozolin), pesticides like DDT (dichlorodiphenyl trichloroethane), methoxychlor, chlorpyrifos, phytoestrogens (genistein and coumestrol), and pharmaceutical agent DES (diethylstilbestrol), are frequently detected in in the food chain according to the National Health and Nutrition Examination Survey (NHANES) biomonitoring data (Diamanti-Kandarakis et al., 2009). Toxic chemicals like these have been identified in human adipose tissue and bodily fluids such as milk, amniotic fluid, urine, and serum. Pregnant women often encounter EDCs through personal care products, household items, and pharmaceuticals (Frye et al., 2012; Kahn et al., 2020; Haggerty et al., 2021). Additionally, women working in chemical industries or farming are particularly exposed to EDCs found in pesticides and herbicides factories or on farms (Combarnous, 2017).

Premature ovarian failure and abnormal sex steroid hormone levels are notable health concerns due to their association with early infertility and an elevated risk of conditions like osteoporosis, depression, cardiovascular disease, and premature mortality (Yoon et al., 2014; Darbre, 2018).

There is considerable apprehension regarding environmental pollutants, encompassing, but not limited to EDCs, and their impact on the female reproductive system. Heightened concerns surround the adverse health consequences of dioxin, recognized as the most potent group of organic persistent pollutants (POPs). Numerous reports have raised serious alarms, indicating that occupational or accidental exposure to dioxins can trigger conditions such as cancer, cardiovascular events, pulmonary diseases, and substantial abnormalities in the female reproductive system (Yonemoto, 2000).

The term “Dioxins” refers to a group of 210 closely related compounds that share structural similarities but exhibit significant variation in toxicity. This group comprises 75 polychlorinated dibenzo-p-dioxins (PCDDs) and 135 polychlorinated dibenzo-p-furans (PCDFs). While most of these compounds do not pose a health hazard at commonly found environmental levels, 17 congeners among them are highly toxic. The 2,3,7,8-tetrachlorodibenzo-p-dioxin (TCDD) stands out as the most toxic congener within the dioxin group. As a prototype of halogenated aromatic compounds, TCDD is frequently employed to illustrate the toxicological effects of dioxins on organisms. Numerous studies have reported that TCDD induces a range of toxicological effects, encompassing reproductive, developmental, and carcinogenic impacts in both exposed individuals and a diverse array of experimental animal models (White and Birnbaum, 2009; Faiad et al., 2022; Aldeli et al., 2023; Faiad et al., 2023; Hammoudeh et al., 2023).

In rats, exposure to even a low concentration of TCDD (less than 50 μg/kg), with a reported half-life of approximately 19 days can induce various toxic effects, including hepatotoxicity, reproductive and developmental toxicity, thymic atrophy, wasting syndrome, immune suppression, and acute lethality (Geyer et al., 2002; Marshall and Kerkvliet, 2010). In humans, the half-life of TCDD is longer, it is estimated to be approximately 8 years. The main reported data on the toxicity of TCDD exposure in humans comes primarily from two sources, the exposure to the agent orange during the Vietnam war as well as the exposure to dioxins by industrial accidents such as what happened in July 1976, when a chemical plant explosion near Seveso in Italy exposed locals to high level of 2,3,7,8-tetracholorodibenzo-p-dioxin, causing a public health crisis to the residential population (Pocchiari et al., 1986; Landi et al., 1998; Eskenazi et al., 2018).

In both cases, a wide range of toxicological effects have been authenticated in the TCDD-exposed individuals as well as in the subsequent generations (Mocarelli et al., 2011). Due to its long half-life, effects of TCDD that are observed up to F2 generation can be refer to direct multigenerational exposure. Exposure the female to (F0) can persist in the body leading to immediate exposure of any offspring (F1), through the placenta and breast milk (Prokopec et al., 2020). However, several studies have shown that the endocrine-disrupting effects of TCDD are, at least in part, caused by a direct action on the ovary, suggesting that TCDD can modify the hormonal profile in the exposed female, in part, by binding to its receptor known as the Aryl hydrocarbon Receptor (AhR) (Hirakawa et al., 2000; Hirakawa and Minegishi, 2006).

The main objective of this review is to succinctly summarize, elucidate, discuss, and ultimately emphasize new perspectives on the toxicological impacts of TCDD on the female reproductive system. To achieve this, a comprehensive examination of the ovarian structure and ovarian steroidogenesis was undertaken. Subsequently, the elusive explanation of the role of the AhR in mediating dioxin toxicity was provided, with particular attention given to its function in the female reproductive system. Additionally, the review delves into and discusses the effects of dioxin on Folliculogenesis and preimplantation embryos, on the hormonal system and its levels, and on endometriosis.

## 2 Structure and functions of ovary

The ovary, a vital female reproductive organ, serving two essential physiological roles. Firstly, it oversees the development, differentiation, and release of oocytes for fertilization, contributing to reproductive processes (Hernández-Ochoa et al., 2009). Secondly, the ovary is responsible for synthesizing and secreting sex steroid hormones, including estrogens, progesterone, and androgens. These hormones play a pivotal role in sustaining follicle development, ensuring fertility, regulating proper menstrual/estrous cyclicity, and supporting pregnancy (Hirshfield, 1991; Hernández-Ochoa et al., 2009). The cycling ovary encompasses ovarian follicles at various developmental stages, and after ovulation, one or more corpora lutea form depending on the species. The transformative process through which the most immature follicles (primordial) evolve into preovulatory follicles is known as Folliculogenesis (Oktem and Urman, 2010).

Primordial follicles, characterized by a singular oocyte enveloped by a solitary layer of flattened somatic cells called “granulosa cells” are limited in number. During Folliculogenesis, these granulosa cells undergo stimulation, transform into a cuboidal shape, and proliferate to form multiple layers around the oocyte. Simultaneously, the oocyte enlarges, and additional somatic cells, known as “theca cells” are recruited to the follicular structure. Ultimately, at the end of Folliculogenesis, the follicle develops a fluid-filled cavity referred to as antrum, marking its transition into an antral follicle. Antral follicles serve as a crucial source of ovarian steroids and have the capability of ovulation upon appropriate stimulation by Luteinizing Hormone (LH), a hormone produced by gonadotropic cells in the anterior pituitary gland. The production of LH is regulated by gonadotropin-releasing hormone from the hypothalamus. In females, an acute rise of LH known as an LH surge, triggers ovulation and development of the corpus luteum. Following ovulation or the release of the oocyte from the follicle, the remaining granulosa and theca cells undergo a differentiation process known as Latinization. This transformation results in the conversion of granulosa and theca cells into luteal cells, and the formerly follicular structure is now recognized as a corpus luteum (CL). A functional corpus luteum produces progesterone, essential for successful implantation and the maintenance of pregnancy. In the absence of fertilization or the achievement of pregnancy, the corpus luteum undergoes a process of cell death termed Luteolysis or corpus luteum retrogression. Disturbances in Folliculogenesis and corpus luteum formation can lead to adverse reproductive outcomes, including anovulation, reduced fecundity, infertility, estrogen deficiency, and premature ovarian failure (Craig et al., 2011; Carlson, 2018).

### 2.1 Ovarian steroidogenesis

The antral follicle and the corpus luteum (CL), both pivotal structures in the ovary, function as steroidogenic glands. Steroidogenesis in the antral follicle has been meticulously examined in both cellular compartments, adhering to the two gonadotropin theory of ovarian steroidogenesis. This theory precisely elucidates the collaborative efforts of granulosa and theca cells in the synthesis of ovarian steroids (Williams and Erickson, 2015).

**FIGURE 1:**
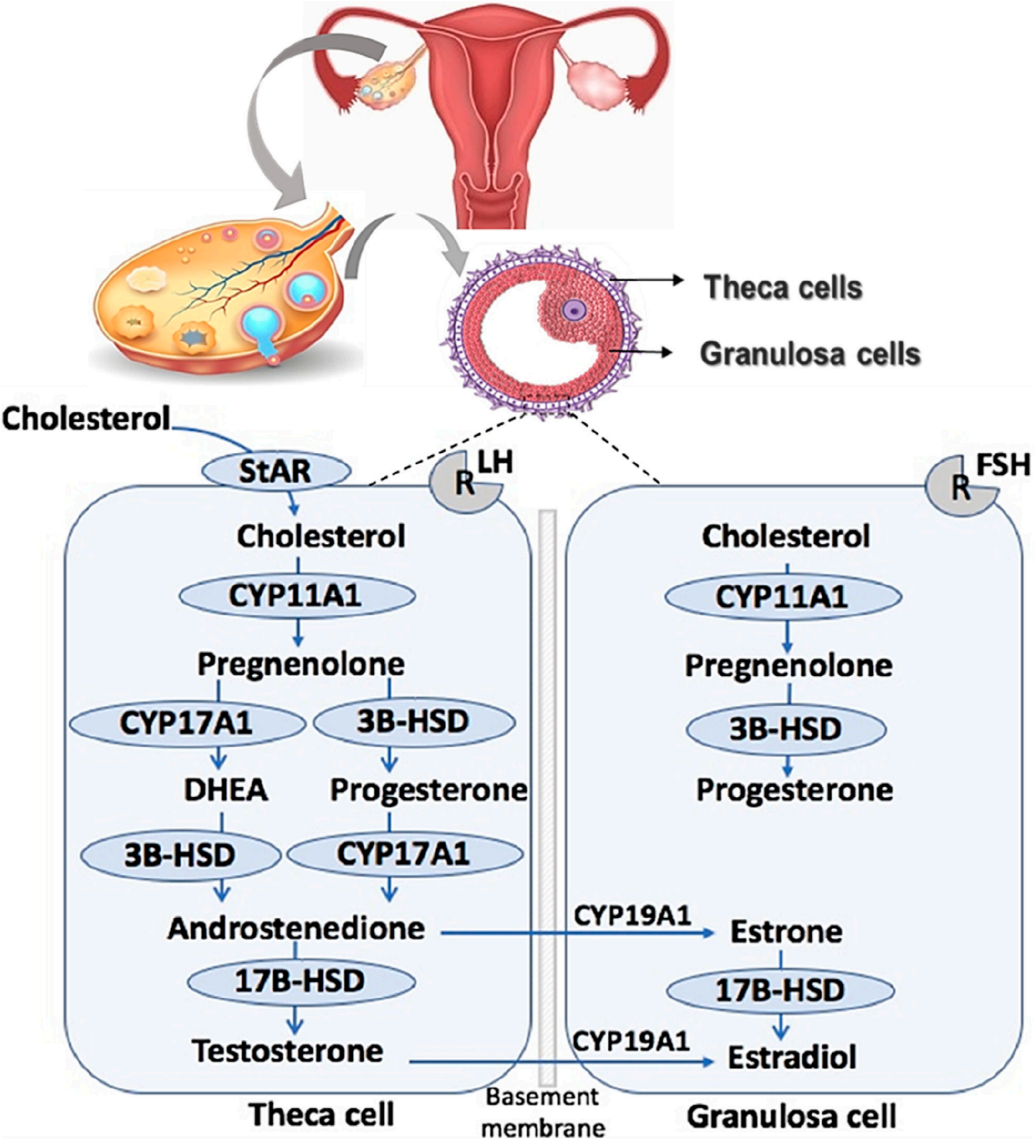
Ovarian steroidogenesis. Ovarian steroidogenesis requires collaborative interactions between the theca and granulosa cells within the follicle. STAR, Steroidogenic acute regulatory protein; CYP11A1, cytochrome P450 11A1 or cholesterol side-chain cleavage; 3β-HSD, 3β-hydroxysteroid dehydrogenase; CYP17A1, cytochrome P450 17A1 or 17a-hydroxylase, 17,20 desmolase; 17β-HSD, 17β-hydroxysteroid dehydrogenase; CYP19A1, cytochrome P450 19A1 or aromatase.

Figure 1 provides a comprehensive overview of ovarian steroidogenesis in both theca and granulosa cells. Theca cells house receptors for luteinizing hormone (LH), released from the anterior pituitary upon binding to its receptor. LH signals are crucial for theca cells, stimulating the transcription of genes encoding enzymes necessary for the conversion of cholesterol into androgens, including androstenedione and testosterone. Conversely, granulosa cells possess receptors for follicle-stimulating hormone (FSH), also released from the anterior pituitary (Craig et al., 2011). Similarly, FSH signals play pivotal roles in granulosa cells by upregulating the expression of genes encoding enzymes that convert theca-derived androgens into estrogens, such as 17β-estradiol (E2) and estrogen (Wu et al., 2022). Cholesterol in the theca cell can be acquired through internalization via lipoprotein receptors or synthesized *de novo* (Arias et al., 2022).

Upon residing in the cytoplasm, cholesterol undergoes transport into the mitochondria facilitated by Steroidogenic Acute Regulatory Protein (STAR) (Christenson and Strauss, 2000; Stocco, 2001). Inside the mitochondria, cholesterol undergoes conversion into pregnenolone through the action of cytochrome P450 cholesterol side-chain cleavage (CYP450scc; CYP11A1) (Craig et al., 2011). Pregnenolone is then translocated from the mitochondria to the smooth endoplasmic reticulum, where it is further converted to progesterone by 3β-hydroxysteroid dehydrogenase (3β-HSD) (Penning, 1997) or to dehydroepiandrosterone (DHEA) by 17α-hydroxylase-17,20-desmolase (CYP45017a; CYP17A1). Both 3β-HSD and CYP17A1 facilitate the diversion of progesterone and DHEA into androstenedione, respectively (Rossetti et al., 2016).

Androstenedione can undergo transformation into testosterone within the theca cell through the action of 17β-hydroxysteroid dehydrogenase (17β-HSD) or be transported into the granulosa cell. In the granulosa cell, aromatase (CYP450arom; CYP19A1) converts androstenedione into estrone, and testosterone is transformed into 17β-estradiol (E2) (Guo et al., 2007). Additionally, within granulosa cells, estrone can be converted into E2 through the action of 17β-HSD (Karman et al., 2012a). E2, the most potent estrogen in female reproduction, undergoes further metabolism into 2-hydroxyestradiol by CYP1A1/2 and CYP3A4 or alternatively into 4-hydroxyestradiol by CYP1B1 (Tsuchiya et al., 2005).

### 2.2 Ovarian hormone receptors

The endocrine system is a complex network of glands that release hormones into the bloodstream, serving as chemical messengers to regulate various physiological functions throughout the body, with a particular impact on the reproductive system (Darbre, 2018). Likewise, the neurosecretory cells, glands, and nonendocrine cells within the neuroendocrine system release hormones into the bloodstream, directly or indirectly influencing the expression of target genes. Lipophilic hormones, such as steroid and thyroid hormones, directly bind to receptors such as the aryl hydrocarbon receptor (AhR), leading to alterations in gene expression (Singh and Verma, 2017; Darbre, 2018).

Hydrophilic hormones, such as peptides or catecholamines, indirectly engage their respective receptors on the cell’s surface. This activation initiates intracellular signal transduction pathways, commonly known as second messenger systems, which subsequently interact with gene expression (Darbre, 2018). Ovarian hormone receptors encompass those specialized for sex steroid hormones like estrogen, progestins, and androgens (Drummond et al., 2002), as well as orphan receptors like the AhR (Pocar et al., 2005), and gonadotropin receptors that bind to LH and FSH. Both sex steroid hormone receptors and AHR function as ligand-attached transcription factors, binding to DNA and regulating the expression of specific genes. On the other hand, LH and FSH receptors are G protein-coupled receptors that modulate various cell functions by initiating second messenger signaling pathways upon binding to their peptide hormone ligand (Althumairy et al., 2020).

Disruption of hormonal functions can occur due to various factors, and among them, an increasing number of environmental contaminants are recognized as endocrine disruptors (EDCs), leading to ovarian toxicity (Costa et al., 2014). In the ovary, the effects of EDCs appear to be linked to estrogen receptors (ESRs), the androgen receptor (AR), and the aryl hydrocarbon receptor (AhR) (Sofo et al., 2015).

#### 2.2.1 Estrogen receptors (ESRs)

Estrogen plays pivotal roles in the female reproductive system, and its biological functions are primarily mediated by two estrogen receptors, namely, ESR1 and ESR2. These receptor subtypes exhibit distinct tissue expression patterns, ligand specializations, and functions (Drummond and Fuller, 2009). Within the ovary, ESR1 is primarily expressed in theca and interstitial cells, and its central role is to regulate steroidogenesis in theca cells (Britt and Findlay, 2002). In contrast, ESR2 is predominantly expressed in granulosa cells, where its fundamental functions include FSH-directed granulosa cell differentiation, follicle maturation, and ovulation (Drummond and Fuller, 2009). Moreover, the transmembrane G protein-coupled receptor 30 (GPR30) was recently identified as having a high affinity for estradiol, leading to its renaming as G protein-coupled estrogen receptor 1 (GPER) (Thomas et al., 2005). Apart from estradiol, this receptor also binds selective estrogen receptor modulators (SERMs), such as tamoxifen, as well as antagonists, such as ICI 182780, eliciting an agonistic response (Thomas et al., 2005). GPER exhibits widespread expression throughout the human body, being present in both normal and pathological tissues (Revankar et al., 2005; Sanden et al., 2011). Specifically, GPER has been detected in ovarian cells and is believed to play crucial physiological and pathological roles in the ovary (Albanito et al., 2007; Kamanga-Sollo et al., 2008). Moreover, GPER expression has been observed in high-risk epithelial ovarian cancer and is associated with poor survival rates (Smith et al., 2009).

#### 2.2.2 Androgen receptor (AR)

The androgen receptor (AR) is present in the ovaries of diverse species, ranging from rodents to primates, including humans. Its expression primarily occurs in granulosa cells and oocytes, with lesser presence in theca/interstitial cells (Pelletier, 2000). While the functions of androgens and the AR are well understood in male reproduction, their roles in females remain less explored. Research on AR knockout mice has revealed the critical importance of AR function in maintaining female fertility. It optimizes follicular growth, eventual follicle development, and ovulation (Walters et al., 2010).

#### 2.2.3 Aryl hydrocarbon receptor (AHR)

**FIGURE 2:**
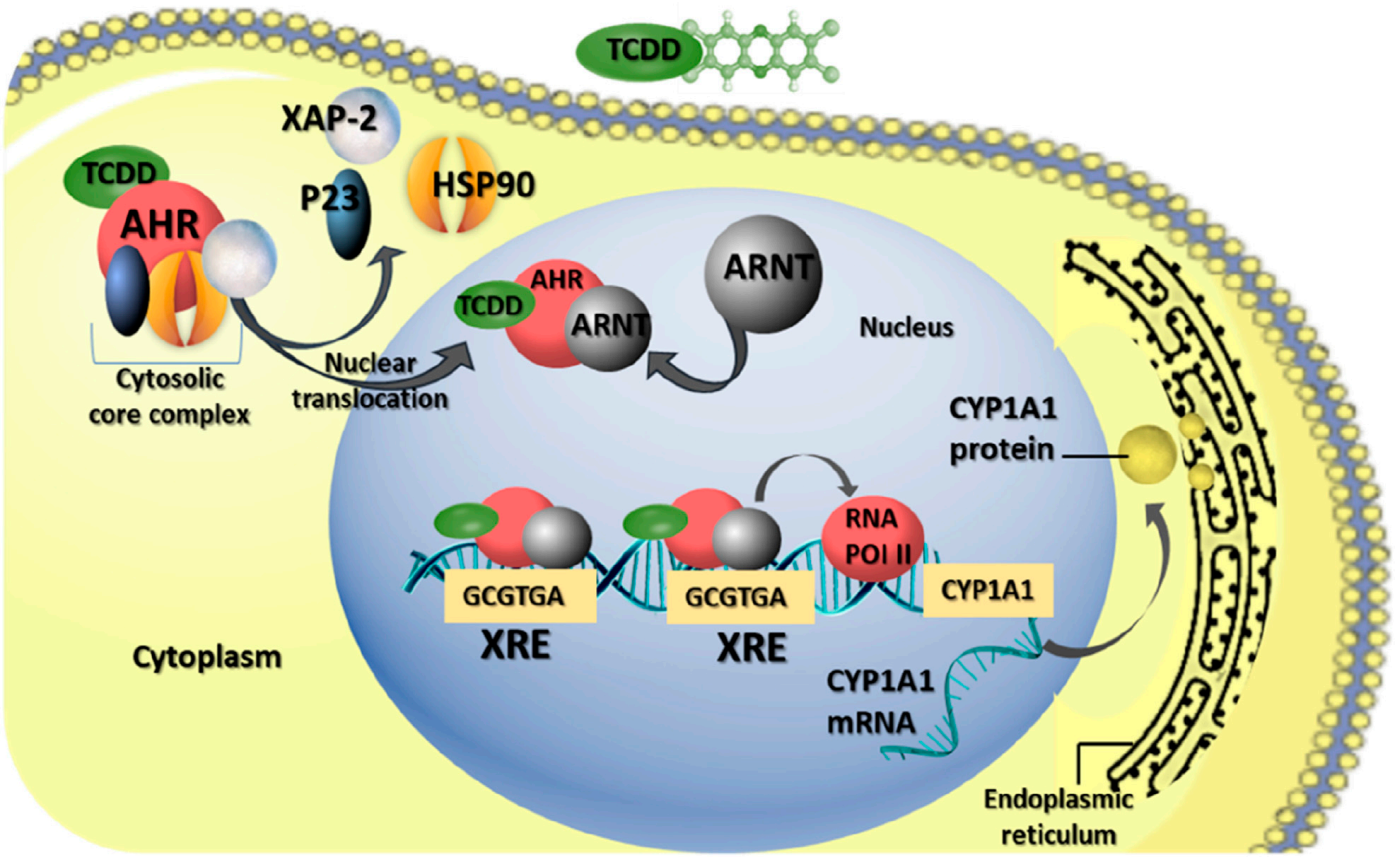
AHR-mediating reproductive toxicity of TCDD in mammalian female. The biological effects of TCDD are mediated through AHR pathways. Upon TCDD binding to AhR, the AhR complex, initially residing in the cytoplasm with chaperone proteins [heat shock protein 90 (HSP90), X-associated protein 2 (XAP-2), and co-chaperone (p23)], undergoes translocation to the nucleus. There, it dimerizes with the aryl hydrocarbon receptor nuclear translocator (ARNT). This AhR-ARNT complex binds to the xenobiotic responsive element (XRE), promoting the activation of various genes, including cytochrome P450 A1 (CYP1A1).

AhR is a ligand-activated nuclear transcription factor widely distributed in vertebrates. It serves as an intracellular regulator of xenobiotic signaling pathways, including those involving man-made chemicals (Hirakawa et al., 2000). Initially identified in toxicological research, AHR mediates the toxicity induced by xenobiotics such as halogenated dibenzo-p-dioxins and similar compounds (Hernández-Ochoa et al., 2009). Under normal conditions, AHR resides in the cytoplasm, forming complexes with at least three distinct chaperone proteins: heat shock proteins 90, immunophilin-like protein XAP2, and co-chaperone p23 (Petrulis and Perdew, 2002). Upon binding a ligand, AHR undergoes a conformational change, leading to its dissociation from the chaperone proteins and subsequent translocation into the nucleus (Heid et al., 2000). Once in the nucleus, the ligand-bound AhR associates with the aryl hydrocarbon nuclear translocator (ARNT) (Heid et al., 2000). The resulting ligand-AHR-ARNT complex binds to specific enhancer sequences known as AHR response elements (AHRE), or xenobiotic responsive elements (XRE), located in the promoter regions of target genes, thereby activating their expression (Hankinson, 2005; Pocar et al., 2005) (Figure 2).

Among the well-studied genes activated by AHR are those belonging to the cytochrome P450 subfamily, such as CYP1A1, CYP1A2, and CYP1B1, which encode xenobiotic-metabolizing enzymes (Barnett et al., 2007a). Notably, the induction of CYP1A1 gene expression serves as a key molecular consequence of exposure to 2,3,7,8-tetrachlorodibenzo-p-dioxin (TCDD), often used as a biomarker for dioxin exposure (Nebert et al., 2000).

#### 2.2.4 Roles of AHR in the female reproductive system

Numerous studies employing diverse techniques such as immunohistochemistry, radio-labeling, RT-PCR, and western blot analysis have demonstrated the expression of the AHR protein and its translocator (ARNT) in various ovarian cell types (oocytes, granulosa cells, and theca cells) across different mammalian species, including primates, humans, pigs, rats, mice, and rabbits. These findings suggest a potential role of AHR in ovarian function (Hernández-Ochoa et al., 2009). In this context, the biological functions of the AhR in the ovary have been explored using AhR-knockout mice (AhRKO). It was observed that AhRKO mice had significantly more fully formed primordial follicles. Further evidence supporting the role of AhR in regulating follicle growth comes from studies examining follicle growth *in vitro* and assessing the proliferation rate of granulosa cells (Benedict et al., 2000). Specifically, research indicates that cultured AHRKO antral follicles exhibit slower growth, as evidenced by a smaller follicle diameter after 168 h of culture compared to wild-type (WT) follicles (Barnett et al., 2007b). Moreover, it was demonstrated that an agonist capable of activating the AhR pathway enhances the proliferation of cultured rat granulosa cells by enhancing the mitogenic effects of follicle-stimulating hormone (FSH) and estradiol (E2) (Bussmann et al., 2006). Additionally, Benedict et al. (2003) investigated DNA fragmentation in ovarian follicles, a key indicator of apoptosis, and found no difference between AHRKO and WT follicles. Collectively, these data suggest that the AHR may regulate follicle growth by promoting granulosa cell proliferation.

It is reasonable to hypothesize that AHR contributes to the regulation of ovarian follicular growth, the capacity of follicles to produce sexual steroid hormones, and the detection of the ovulation process and corpus luteum (CL) formation. The synthesis of steroid hormones is predominantly regulated by the anterior pituitary gland and occurs sequentially in the theca and granulosa cells within follicles (Drummond et al., 2002; Baba et al., 2005).

## 3 Dioxins and their sources

Dioxin is a term used to describe a group of closely related compounds with similar chemical structures but which vary greatly in their toxicity. Dioxin comprise polychlorinated dibenzo-*p*-dioxins (PCDDs), polychlorinated dibenzofurans (PCDFs) and co-planar polychlorinated biphenyls (PCBs). The general chemical structures of PCDD and PCDFs consist of two benzene rings connected by one or two oxygen atoms and can contain four to eight chlorines (Van den Berg et al., 2006). Due to their extreme lipophilicity, dioxins can bio accumulate by both plants and animals, causing thus several developmental abnormalities in (Hanano et al., 2014a; Hanano et al., 2014b; Hanano et al., 2015; Hanano et al., 2018a; Hanano et al., 2018b; Hanano et al., 2018c; Hanano et al., 2019; Mahfouz et al., 2020a; Mahfouz et al., 2022). Terrestrial and aquatic environments are objected to dioxin contamination, subsequently elevated levels of dioxins can be accumulated in marine organisms and livestock. From these sources dioxins can readily get in human food chains and thereby constitute a potentially serious health risk (Desforges et al., 2016; Hanano et al., 2018a). Dioxins are realized into the environment through natural sources such as volcanic activities or forest fires (Li et al., 2006a). Such episodes are becoming more common with particular increases in the incidence of large-scale forest fires over two last decades (Zhang et al., 2016; Oliveira et al., 2020). In addition, dioxins are also release into the environment by certain industry including the synthesis of chlorinated aromatic and aliphatic compounds, such as pesticide and herbicides (Chen et al., 2005) and paper production (Haq and Raj, 2020). Other unintentional sources of dioxins include wastes containing chlorinated aromatic combination from chemical facilities, sewage sludge, incineration of domestic and medical wastes, incineration of fossil fuels and fly ash storage (El-Shahawi et al., 2010). Lastly, road transport emissions and plastic substances have also been identified as an important sources of different kinds of Dioxins (Mahfouz et al., 2020b). In term of toxicity, it is well recognized that dioxin at low dose may seriously disrupt reproduction in humans and animals (Li et al., 2006a). It decreases fertility, increases prenatal mortality, causes birth defects, and increases the peril of endometriosis (Vandenberg et al., 2013; Yilmaz et al., 2020). However, at high dose, dioxin suppresses the immune system (Marshall and Kerkvliet, 2010) and causes weight lack, oxidative stress, lymphoid atrophy, gonadal atrophy, and cutaneous lesions (Giampaolino et al., 2020). In addition, dioxins motivate certain kinds of cancers and disrupts reproduction (Li et al., 2006a). When it comes to the mean dioxin intake and exposure dose limits, international agencies have indeed set a “safe” or tolerable daily dose for dioxin. In 1994, the EPA defined a dose of 0.01 pg TEQ/kg body weight/day, which is equivalent to 0.7 pg/day for a 70 kg adult, as posing a cancer risk of one additional cancer in one million people exposed (US EPA Environmental Protection Agency, 1994). This “risk dose equivalent” is primarily designed to protect adults and does not include any added protection for children. In 1990, the WHO established a tolerable daily intake (TDI) for dioxin, ranging from 1 to 4 pg/kg body weight/day, corresponding to a daily ingestion of 70–280 pg in a 70 kg adult (WHO, 1998). Additionally, the Agency for Toxic Substances and Disease Registry assessed the non-cancer risks from dioxin exposure by setting minimal risk levels (MRLs) for acute, sub-chronic, and chronic exposures to dioxins. The chronic MRL was established based on dioxin’s developmental neurotoxicity in rhesus monkeys and was set at 1 pg/kg body weight/day (Stellman et al., 2003).

### 3.1 Effects of TCDD on mammalian females

Due to its lipophilic nature and slow metabolism, TCDD has a prolonged environmental half-life and can persist in humans for over 10 years following a single exposure (Karman et al., 2012b). TCDD has been detected in human adipose tissue, blood serum, breast milk, and ovarian follicular fluid (Humblet et al., 2011; Ulaszewska et al., 2011). Recognized as one of the most potent carcinogenic compounds, TCDD exerts significant toxic effects on various tissues and organs (Karman et al., 2012a). Regarding reproductive toxicity, previous research has demonstrated that TCDD directly and indirectly affects ovarian functions, leading to disruptions in the estrous cycle (Brown et al., 1998). TCDD exposure has been associated with delayed puberty and early onset of menopause in women (Warner et al., 2007). Similarly, in female rodents, TCDD exposure results in early puberty, irregular estrous cycles, reduced or blocked ovulation, decreased circulating estradiol levels (E2), and premature reproductive senescence (Franczak et al., 2006; Jablonska et al., 2010). Given that disruption of steroid hormone synthesis, activity or metabolism can lead to follicular dysfunction and atresia, thereby affecting the functions of female reproductive system (Sanderson, 2006), female exposure to TCDD influences the production of estrogens and progesterone by porcine granulosa cells (Jablonska et al., 2011; Jablonska et al., 2014). In this regard, it was reported that TCDD disrupted endocrine signaling pathways, potentially causing significant impairments in growth and reproduction (Baldridge et al., 2015). Mechanistically, this effect was induced by altering the expression of transcripts involved in follicular atresia, as well as in cell proliferation and cell cycle regulation (Ruszkowska et al., 2018; Nynca et al., 2019). Further investigations have focused on the impact of TCDD on enzymatic activities within the estrogen biosynthesis pathway. It has been observed that TCDD directly affects antral follicles and inhibits the production of steroid hormones (Karman et al., 2012a). Table 1 summarizes the information on the models, species, molecules used and effects of dioxins on mammalian females.

**TABLE 1:** Summarizes the information on the models, species, molecules used and effects of dioxins on females.

Models	Species	Dose/age	Compound	Route of exposure	Toxic effects	Reference
Rat	Holtzman	Maternal exposure on GD15 and GD8/(1 μg/kg)	TCDD	Oral dose	GD15 Decreased ovarian weights, abnormalities in the reproductive organs	Gray et al. (1995)
					GD8 Decreased fertility, increased incidence of persistent estrus, and decreased ovarian size	
Rat	Sprague-Dawley	Immature females/(10 μg/kg)	TCDD	Oral dose	Inhibition of ovulation; and an increase in the concentration of estrogen	Petroff et al. (2000)
Rat	Sprague-Dawley	Maternal exposure on GD13/(0, 0.04, 0, 2, 1 μg/kg)	TCDD	Oral dose/maternal	Reduced body weights, Plasma estradiol levels in dams and female pups were reduced and FSH levels were increased in female pups. Decreased in isolated ovarian follicles	Myllymäki et al. (2005)
				Exposure		
Mice	C57BL/6 females	Maternal exposure on GD15/(10 μg/kg)	TCDD	Oral dose/maternal	Decreased of Placental weights and pups birth weights, and developmental exposure of either parent to TCDD is associated with preterm birth in a subsequent adult pregnancy due to altered progesterone expression and placental inflammation	Ding et al. (2011)
				Exposure		
Zebrafish	*Danio rerio*	F0 Generation	TCDD	1 h, static waterborne at 3 and 7 wpf^a^	Changed in sex ratio	Baker et al. (2014)
		50 pg/mL			Increased skeletal malformations	
Rat	Wistar	Maternal exposure on GD15/(0,5, 1, 2 mg/kg)	TCDD	Oral dose/maternal	A decrease in the mating index, fertility, and the average number of embryos implanted and nested in the uterus	Mai et al. (2020)
				Exposure/		
Rat	Sprague-Dawley	Maternal exposure on GD (8–14)/(100 or 500 ng/kg)	TCDD	Oral dose/maternal	Reduced the ovarian reserve and inhibited follicular development in adult female offspring, an effect that persisted for multiple generations	Yu et al. (2020)
				Exposure/		
Mice	BALB/c	Adult female (25 μg/kg)	TCDD	Oral dose	Atrophy of the ovary, histological alterations in ovary. Female fertility was declines across generations with a reduced male\female ratio	Aldeli et al. (2023)
		Maternal exposure on GD (13)/(25 μg/kg)				

### 3.2 Effect of TCDD on folliculogenesis and preimplantation embryos

Several studies have revealed inconsistencies in the effects of TCDD on folliculogenesis and preimplantation embryos. Son et al. (1999) conducted a study on pre-pubertal IHR rats following oral administration of TCDD, which resulted in altered gene expression of the CYP1A1 gene but no change in the expression of the CYP1B1 gene. This was associated with a significant decrease in the rate of ovulation and the number of developing follicles in the ovary (Son et al., 1999).

Yokoi et al. (1993) demonstrated that assessing the gene expression of AHR, ARNT, and CYP1A1 in TCDD-exposed embryos at different stages could help identify stage-specific effects of TCDD on preimplantation embryos. Conversely, Wu et al. (2002) found that TCDD exposure did not alter cell proliferation, differentiation, or survival in embryos. Similarly, Blankenship and co-workers reported that TCDD stimulated blastocyst differentiation without affecting embryo viability or the number of embryo cells (Blankenship et al., 1993).

Several studies also indicated that the sensitivity of each stage of embryonic development of rats differs to TCDD. The changes in gene expression of *CYP1A1*, *AhR* and *Arnt* genes at specific stages of life of fetuses of rat mothers were evaluated as a response to TCDD, namely, the single-cell stage (fertilized egg), the two-cell stage, the eight-cell stage and the blastocyst stage, and it was found that at single-cell and two-cell stages, the expression of these genes did not change, while the expression of *CYP1A1* increased in the eight-cell stage, while no change was observed in the blastocyst stage (Penning, 1997).

Another study revealed that injecting TCDD compound into female mice can directly impact the embryo’s implantation stage, leading to reduced viability. Embryos from injected mothers exhibited poorer survival rates compared to those from control mothers (Hanukoglu, 1992). This finding was corroborated by research involving pregnant NIH mice exposed to increased TCDD doses from days 1–8 of gestation. The study demonstrated a decrease in the number of implantation embryos when dosed on days 1–3 of pregnancy, just before the implantation stage, compared to mothers dosed on days 4–8. This highlights the heightened sensitivity of endometrial implantation areas during early developmental stages (pre-implantation) to TCDD compared to the post-implantation stage (Tsuchiya et al., 2005).

Additionally, administering TCDD orally to pregnant rats of the same strain with doses of 100 or 500 ng/kg from gestation days 8–14 resulted in poor ovulation and a decrease in the number of ovarian follicles, particularly primary and secondary follicles in females of the second and third generation (Britt and Findlay, 2002). Karman et al. (2012c) suggested that species’ sensitivity to TCDD could be linked to their biological capacity to metabolize TCDD and the level of AHR expression. While the effects of TCDD on folliculogenesis vary by species, exposure to TCDD has been shown to decrease or block ovulation in rodents *in vivo* (Jung et al., 2010). The mechanism by which TCDD blocks ovulation likely involves reducing the numbers of granulosa cells in the S-phase and inhibiting the levels of cyclin-dependent kinase 2 (Cdk2) (Jung et al., 2010). Furthermore, TCDD exposure was reported to diminish ovarian reserve in rats and impede follicular development in adult female offspring, effects observed across the F1 and F2 generations. Changes in ovarian Anti-Mullerian hormone (AMH) levels may contribute to these adverse effects, shedding light on the multigenerational impacts of TCDD on follicular development and ovarian quality (Yu et al., 2020).

### 3.3 Effect of TCDD on the hormone system

Studies have documented that TCDD alters the steroidogenic structure of both granulosa and theca cells, likely mediated by a cholesterol-transporter protein found within the mitochondria known as steroidogenic acute regulatory protein (STAR) (Murray, 2001). Several studies have highlighted the negative effects of dioxin in inhibiting estrogen biosynthesis, which is considered one of the most significant hormones in the female reproductive system (Petroff et al., 2001). In rats, TCDD exposure reduces the number of antral follicles without any marked changes in atresia, suggesting that TCDD has an antiproliferative effect on the rat ovary (Heimler et al., 1998). In the same context, Li et al. (2006b) reported that TCDD at very low concentrations (2 ng/kg) significantly reduced serum progesterone levels but had no effect on serum estradiol.

Furthermore, the level of TCDD in the uterus was found to be similar to its levels in the liver but lower than in adipose tissue. These findings suggest that TCDD sensitivity may be influenced by its localized accumulation in the uterus (Li et al., 2006a). Additionally, immature hypophysectomized rats (IHR) exposed to TCDD exhibited significant ovarian dysfunction, leading to blocked ovulation (Son et al., 1999). Karman et al. (2012c) suggested that the effects of TCDD on ovarian steroidogenesis are likely attributed to its inhibitory effects on specific steroidogenic enzymes such as Hsd17b1 and Cyp19a1, resulting in steroidogenic defects in antral follicles. Moreover, exposure of mice to 90 ng TCDD/kg/day markedly increased the testosterone-to-estradiol ratio (Maranghi et al., 2013), while TCDD exposure in chicken ovaries led to decreased estradiol levels (Sechman et al., 2014). Moreover, Grochowalski et al. (2000) demonstrated that treatment of porcine thecal and granulosa cell co-cultures with 0.1 nM or 10 nM TCDD resulted in reduced estradiol levels, while progesterone levels were significantly decreased with 10 nM TCDD treatment. Similarly, isolated mouse antral follicles exposed to TCDD (0.1–100 nM) showed reduced levels of progesterone, androstenedione, and estradiol (Karman et al., 2012c; Karman et al., 2012d). These hormonal deficiencies were restored with the addition of pregnenolone, suggesting that TCDD may act prior to pregnenolone formation to decrease hormone levels (Karman et al., 2012b). Consistent with this, female mice exposed to TCDD exhibited low levels of estradiol (Huang et al., 2011). A similar scenario was observed in Sprague-Dawley rats after oral administration of TCDD for a long period (Chen et al., 2009). Other studies have revealed that TCDD inhibits estrogenic signaling in the kidney and liver but paradoxically promotes estrogenic signaling in the pituitary gland within the same individual (Yoshida et al., 2020). In conclusion, the effects of dioxin on the female reproductive system have shown considerable variability in the literature, likely attributed to differences in TCDD concentration, duration of exposure, animal species, and age of the animal. Understanding the impact of dioxins on the ovary is crucial for refining existing policies aimed at addressing dioxin-induced ovarian toxicity. This knowledge can also facilitate the development of novel therapies to address dioxin-induced abnormalities in both reproductive and non-reproductive health (Patel et al., 2015).

### 3.4 Effect of TCDD on endometriosis

Endometriosis is characterized by the presence of stromal and/or endometrial glandular epithelium implants outside the uterine cavity, often leading to symptoms such as pelvic pain, dysmenorrhea, dyspareunia, urinary symptoms, and sometimes presenting asymptomatically (Soave et al., 2015). Normally, the immune system clears endometrial tissue from the peritoneal cavity. However, dioxin-like environmental pollutants have been implicated in altering the inflammatory processes responsible for tissue clearance, thereby promoting the development of endometrial tissue (Soave et al., 2015). As a marker for endometriosis, the expression of matrix metalloproteinase (MMP) system, which degrades extracellular matrices, has been investigated (Alali et al., 2018). In healthy tissue, progesterone typically suppresses the expression of MMPs (Soave et al., 2015). However, exposure to TCDD can disrupt this balance in endometrial tissue, leading to elevated production of matrix metalloproteinases (MMPs) (Soave et al., 2015). Additionally, TCDD exposure can reduce the levels of progesterone, further exacerbating the increased expression of MMPs in the endometrium (Bruner-Tran et al., 2008).

## 4 Conclusion and future remarks

The current review provides an overview of the effects of dioxin, primarily functioning as endocrine-disrupting chemicals (EDCs), on the female reproductive system at molecular, biochemical, and histological levels, thereby impacting the overall health and function of reproductive organs in mammalian females. Our analysis emphasizes the variability in data related to dioxin’s reproductive toxicity, which is influenced by factors such as dosage, animal species, and age. It is now well-established that the reproductive toxicity of dioxin is predominantly mediated by the AhR. However, other molecular mechanisms remain under investigation. Particularly noteworthy are the regulatory roles of microRNAs (miRNAs) in targeting and suppressing mRNAs related to hormone regulation. Further exploration of these mechanisms is crucial for a comprehensive understanding of dioxin-induced reproductive toxicity and for the development of targeted interventions.
